# Effect of Location, Disinfection, and Building Materials on the Presence and Richness of Culturable Mycobiota through Oligotrophic Drinking Water Systems

**DOI:** 10.3390/jof9111086

**Published:** 2023-11-06

**Authors:** Monika Novak Babič, Gregor Marolt, Jernej Imperl, Martin Breskvar, Sašo Džeroski, Nina Gunde-Cimerman

**Affiliations:** 1 Biotechnical Faculty, University of Ljubljana, 1000 Ljubljana, Slovenia; nina.gunde-cimerman@bf.uni-lj.si; 2 Faculty of Chemistry and Chemical Technology, University of Ljubljana, 1000 Ljubljana, Slovenia; gregor.marolt@fkkt.uni-lj.si (G.M.); jernej.imperl@fkkt.uni-lj.si (J.I.); 3 Department of Knowledge Technologies, Jožef Stefan Institute, 1000 Ljubljana, Slovenia; martin.breskvar@ijs.si (M.B.); saso.dzeroski@ijs.si (S.D.); 4 DZR, Luize Pesjakove ulica 11, 1000 Ljubljana, Slovenia

**Keywords:** chemical composition of water, chlorination, drinking water, environmental factors, fungal abundance, fungal richness, human health, materials, ultrafiltration

## Abstract

Safe drinking water is a constant challenge due to global environmental changes and the rise of emerging pathogens—lately, these also include fungi. The fungal presence in water greatly varies between sampling locations. Little is known about fungi from water in combination with a selection of materials used in water distribution systems. Our research was focused on five water plants located in the Pannonian Plain, Slovenia. Sampled water originated from different natural water sources and was subjected to different cleaning methods before distribution. The average numbers of fungi from natural water, water after disinfection, water at the first sampling point in the water network, and water at the last sampling point were 260, 49, 64, and 97 CFU/L, respectively. Chlorination reduced the number of fungi by a factor of 5, but its effect decreased with the length of the water network. The occurrence of different fungi in water and on materials depended on the choice of material. The presence of the genera *Aspergillus*, *Acremonium*, *Furcasterigmium*, *Gliomastix*, and *Sarocladium* was mostly observed on cement, while *Cadophora*, *Cladosporium*, *Cyphellophora*, and *Exophiala* prevailed on metals. Plastic materials were more susceptible to colonization with basidiomycetous fungi. Opportunistically pathogenic fungi were isolated sporadically from materials and water and do not represent a significant health risk for water consumers. In addition to cultivation data, physico-chemical features of water were measured and later processed with machine learning methods, revealing the sampling location and water cleaning processes as the main factors affecting fungal presence and richness in water and materials in contact with water.

## 1. Introduction

Freshwater sources captured in polar ice caps, groundwater, and surface water are of crucial importance for life on our planet. Understanding the mechanisms of their availability, monitoring their quality, studying their effect on health, and taking care of their conservation are thus the most important goals nowadays [1]. The quality of water is strongly affected by the availability of primary water sources, including groundwater, surface water, and rainwater, which differ among countries worldwide [2]. Slovenia is located between central Europe to the north and the Balkan peninsula to the south. The geology of the country is thus very diverse and includes aquifers with different amounts of available groundwater [3]. Eighty-five percent of drinking water is produced from groundwater sources and 3% from rivers, while the rest of the population uses their private wells [4,5]. Due to the extensive use of natural resources, agriculture, and industrialization, most of the natural water needs cleaning and disinfection before being suitable for human consumption [6]. These include aeration, flocculation, sedimentation, and ultrafiltration. Disinfection is mostly carried out with chlorine but also with UV light and ozone [5,6]. Often, combinations of different procedures are used to reach the limits of microbiological parameters set in the Drinking Water Directive. The Recast of the Drinking Water Directive (DWD 2020/2184) includes parameters established in DWD 98/83/EC as *Escherichia coli*, intestinal enterococci, coliform bacteria, *Clostridium perfringens*, and colony count at 22 °C. Novel parameters, such as the number of somatic coliphages and *Legionella*, were added, while *Pseudomonas aeruginosa* and colony count at 37 °C were removed [7]. Although reported in many studies of groundwater, surface water, and drinking water across Europe, fungi are not included as a parameter in the Drinking Water Directive [2,7]. However, their emerging presence in the environment affected the decision of the Commission Expert Group on the Implementation of the Drinking Water Directive (EMEG), which included fungi as a risk parameter for drinking water in hospitals [8]. Although the connection between waterborne fungi and their effect on health is still scarcely investigated, fungi may indirectly cause intoxication through prolonged exposure to their mycotoxins [9,10]. The presence of fungi at consumers’ taps is not affected only by the natural water source and the choice of water cleaning process. After water enters the distribution systems, building materials become a variable factor, adding to the final water quality [11]. This, however, is partly regulated in terms of the physico-chemical suitability of materials, but their effect on biofilm formation is yet to be determined [7]. The selective pressure of materials on fungal abundance and richness in biofilms is scarce [12,13,14]. Fungi, such as *Aspergillus*, *Chaetomium*, *Mucor*, *Penicillium*, and *Sporothrix,* have been isolated from cement and its composites [15]. The genera *Candida*, *Cystobasidium*, *Exophiala*, *Meyerozyma*, *Naganishia*, *Rhodotorula*, *Phialophora*, and *Rhinocladiella* are often isolated from plastics, rubbers, and silicones from water facilities, public places, and private homes [2], while *Penicillium*, *Cladosporium*, *Russula*, and *Tricholadium* colonize metals [16]. Their presence on materials has often been associated with altering materials’ quality and consequently affecting water odor, taste, and color [12,17]. The main mechanisms include mechanical and chemical biodegradation of material via the production of acids and mycelial growth, as previously studied for *Alternaria*, *Aspergillus*, *Cephalosporium*, *Cladosporium*, *Mucor*, *Penicillium*, *Rhizopus*, and *Trichoderma* [17,18].

A previous Slovenian study on cultivable fungi from groundwater and drinking water at consumer taps revealed *Aspergillus*, *Aureobasidium*, *Exophiala*, *Candida*, and *Rhodotorula* as the main water-transmitted genera. It also indicated the possible effects of water cleaning procedures on certain fungi, such as *Aureobasidium*. Fungi have been associated with the chemical composition of water, in particular the presence of calcium, magnesium, and nitrate [19]. The study conducted on water originating from karstic aquifers discussed cultivable water-borne fungi as contaminants in drinking water storage tanks. The choice of material had a significant effect on the number and abundance of fungi. While *Exophiala*, *Furcasterigmium*, *Gloeotinia*, and *Sarocladium* prevailed on the surface of cementious water storage tanks, chlorination proved to be effective against elevated numbers of fungi in water due to poor choice of material [17]. So far, no study in Slovenia has focused on cultivable fungi, following their path in water, or on the effects of materials from the natural source through the drinking water network. Diverse natural features can give a unique insight into fungal populations in relation to different environmental and anthropogenic factors. The current research focused on a previously poorly investigated area within the Pannonian Plain with extensive agriculture nearby. We sampled water at five water production plants using different natural water sources and different processes to obtain clean drinking water. During this study, we focused on physico-chemical features that could possibly affect the presence and richness of cultivable fungi. We further investigated the effectiveness of water cleaning and disinfection against fungi and sampled materials in contact with water at different stages throughout the water network to reveal possible selective preferences for fungal colonization of building materials. Machine learning analysis was used to reveal the most important factors influencing cultivable mycobiota in water and on materials.

## 2. Materials and Methods

### 2.1. Sampling of Water and Surfaces of Materials in Contact with Water

Sampling was conducted according to SIST ISO 5667-5:2007 at five water plants located between two Slovenian rivers, namely Mura in the north-east and Drava in the south-west: Ormož, Lukavci, Mota, Terbegovci, and Podgrad-Segovci. At each water plant, sterile containers were used to collect 10 litres of natural water (NW), water after ultrafiltration (UF) and chlorination (CH), water at the distribution point closest to the water plant (FP), and water at the end of the water network (LP). At the same sampling spots, biofilms from materials in contact with water were also collected, including cement (C), metal (M), rubber (R), and plastic parts (P). Biofilms were scraped in five repetitions from the standardised surface of 1 cm^2^ using sterile cotton swabs (Golias, Ljubljana, Slovenia).

### 2.2. Physico-Chemical Analyses of Water

Temperature, pH, conductivity, electric potential, total dissolved solids (TDS), resistivity, and concentration of oxygen in water samples were measured using a Multimeter PCD6 (Thermo Fisher Scientific, Waltham, MA, USA). The determination of chemical parameters included measurements of water hardness and anion analysis by high-performance ion-exchange chromatography (HPIC), as well as elemental analysis by ICP-MS. The water hardness of the samples was determined by the complexometric titration procedure using a standardized 0.0178 mol/L EDTA solution and complexometric indicators, namely Eriochrome Black T and Murexide, for the determination of the total (°*dH*_TOT_) and calcium (°*dH*_Ca_) hardness, respectively. The carbonate (°*dH*_Carb_) hardness was determined using the standardized 0.100 mol/L HCl solution and an automatic titrator, Metrohm 799 GPT Titrino (Herisau, Switzerland), equipped with a 20.0 mL burette and a combined glass pH electrode (Metrohm 6.0234.100, pH 0–14). The magnesium (°*dH*_Mg_) and non-carbonate (°*dH*_Non-carb_) hardness were calculated subsequently, based on the relation (Equations (1) and (2)) of previously determined values by titration procedures:

°*dH*_Mg_ = °*dH*_Tot_ − °*dH*_Ca_ (1)

°*dH*_Non-carb_ = °*dH*_Tot_ − °*dH*_Carb_ (2)

The analysis of anions in water samples was performed by ion-exchange chromatography after a preliminary purification of the samples with filtration using a 0.45 µm syringe filter (Whatman). The samples were measured by the Thermo Scientific Dionex ICS-5000 Ion Chromatography System (Dionex, Sunnyvale, CA, USA), which consisted of a gradient pump, autosampler AS-5000 with a 20 µL injection loop, conductivity detector (CD), continuously regenerated electrochemical suppressor ADRS 600 (4 mm), and analytical column IonPac AS14A (4 × 250 mm), which was used for the separation of selected analytes (including chloride, nitrite, nitrate, sulphate, and phosphate) using a gradient elution program between 20 and 65 mM KOH eluent and a flow rate of 1.5 mL/min, resulting in a backpressure of approx. 2500 psi and a LOQ value around 0.2 mg/L, depending on the selected analyte.

The elemental analysis was performed using a quadrupole inductively coupled plasma mass spectrometer (ICP-MS Agilent 7900ce, Agilent Technologies, Palo Alto, CA, USA) with the use of internal standards and optimal instrumental parameters, ensuring the lowest detection limits. A forward RF power of 1.5 kW was used with Ar gas flows: carrier 0.85 L/min, makeup 0.28 L/min, plasma 1.0 L/min, cooling 15 L/min, and sample flow rate 0.2 mL/min, measuring one point per mass and acquiring the following isotopes: ^23^Na, ^24^Mg, ^27^Al, ^39^K, ^44^Ca, ^47^Ti, ^55^Mn, ^56^Fe, ^60^Ni, ^63^Cu, ^66^Zn, ^88^Sr, ^137^Ba, ^208^Pb. Prior to the measurement, the samples were diluted 50 times (200 µL/10 mL) using a 1% HNO_3_ solution prepared in ultrapure Milli-Q water (resistivity > 18.2 MΩ·cm). Simultaneously, the sample mass was measured using an analytical scale (Sartorius Entris 224i-1S, Goettingen, Germany) in order to calculate the final results in mass percentage (given in ppm). The calibration curves were based on 9 calibration multi-standards in the concentration range 0.1–1000 µg/L, prepared by the dilution of CRM multi-standard solution periodic table mix 1 for ICP, TraceCERT^®^, Sigma-Aldrich, Hamburg, Germany. 

### 2.3. Fungal Cultivation and Permanent Storage of the Strains

Ten aliquots of 100 mL were taken from each water sample and were filtered through Millipore (Merck, Darmstadt, Germany) filters with a pore diameter of 0.45 µm. Filters were aseptically placed on the media suggested by Novak Babič et al. [2], namely Dichloran Rose-Bengal Agar with the addition of chloramphenicol (DRBC) (Biolife, Monza, MI, Italy) and Sabouraud Glucose Agar (SABG) (Biolife, Monza, MI, Italy) supplemented with penicillin and streptomycin (200 mg/L) to minimize bacterial growth. 

Five repetitions of each swab sample taken from the surface of materials were spread and plated onto the same media as described above. All plates were incubated at 15, 25, and 37 °C for 3 to 14 days. Identical colonies were counted, and colony-forming units per liter of water (CFU/L) and cm^2^ of material (CFU/cm^2^) were calculated as the average number of repetitions.

Pure fungal cultures were obtained from all diverse fungal colonies transferred to the fresh Malt Extract Agar (MEA) medium and incubated for up to 7 days. All pure cultures were permanently stored in a genetically stable form at the Ex-Culture Collection (Infrastructural Centre Mycosmo, MRIC UL, Slovenia (http://www.ex-genebank.com/, accessed on 5 November 2023)), at the Department of Biology, Biotechnical Faculty, University of Ljubljana.

### 2.4. Taxonomical Classification of Isolated Fungal Strains

After the cultures were grown for 3 to 5 days on MEA, the DNA of filamentous fungi was extracted using mechanical lysis [20], while yeast DNA was extracted with PrepMan Ultra reagent (Applied Biosystems, Foster City, CA, USA). All DNA samples were stored at −20 °C prior to use in PCR. Basic presumptive identification of all strains was performed after amplification of the whole internal transcribed spacer region (ITS = ITS1, 5.8S rDNA, ITS2) using primers ITS5 and ITS4 [21]. According to the obtained results, we additionally amplified the sequences of the large subunit rDNA (LSU = partial 28S rDNA, D1/D2 domains; primers NL1 and NL4 [22]) for yeasts, the partial actin gene (*act*; primers ACT-512F and ACT-783R [23]) identifying *Cladosporium*, the partial beta-tubulin gene exons and introns (*benA*; primers Ben2F and Bt2b [24]) for *Aspergillus* and *Penicillium*, and the partial translation elongation factor 1-alpha (*tef*; primers EF1 and EF2 [25]) for *Bisifusarium*. 

Sequences were provided by Microsynth AG (Wien, Austria), assembled by FinchTV 1.4.0 (Geospiza, PerkinElmer Inc., Seattle, WA, USA), and collected for taxonomical comparison in the Molecular Evolutionary Genetics Analysis (MEGA) software version 7.0 [26]. A brief identification of sequences was performed after comparison in the BLAST database (NCBI, Bethesda, MD, USA) [27]. The final taxonomical classification was conducted by the MEGA 7.0 alignment with the corresponding sequences of the type strains from the fungal taxonomical databases Westerdijk Fungal Biodiversity Institute (Utrecht, The Netherlands) and Index Fungorum (www.indexfungorum.org; accessed on 5 November 2023) as follows: sequences of 100 taxonomically related strains and type strains were uploaded along with sequences of isolates from the present study in fasta format into MEGA 7.0. Sequences were aligned with Clustal W software Version 2.1 and manually trimmed to eliminate poorly aligned sites. The data were saved as an alignment in mas format. The evolutionary history was inferred by using the Maximum Likelihood method based on the Tamura-Nei model. The tree with the highest log likelihood was shown. The initial tree(s) for the heuristic search were obtained automatically by applying the Neighbor-Join and BioNJ algorithms to a matrix of pairwise distances estimated using the Maximum Composite Likelihood (MCL) approach and then selecting the topology with a superior log likelihood value. The trees were drawn to scale, with branch lengths measured in the number of substitutions per site [26]. The presumed species of isolates were determined based on the closest type strain relative to the tree. The identified sequences of representative strains from this study were deposited in the GenBank database (NCBI, Bethesda, MD, USA).

### 2.5. Relating Environmental Factors to the Presence of Fungi Isolated from Water and Biofilm 

The collected data were divided into two groups. The first included environmental data, such as water plants’ locations, sample (water or biofilm from material), water type (natural water, water after ultrafiltration, chlorinated water, water at first sampling point in water network, water from retention tanks coated with cement or Xypex, and water at last sampling point in water network), material type (cement, cement coated with Xypex, rubber, metal, and plastics), and physico-chemical properties of water (temperature (°C), pH, conductivity (µS/cm), O_2_ concentration (ppm), electric potential (mV), TDS (ppm), resistivity (Ω/cm^2^), total water hardness, calcium hardness, magnesium hardness, carbonate hardness, non-carbonate hardness (all in °d), and concentrations of Mg^2+^, Ca^2+^, Na^+^, K^+^, Sr^2+^, Ba^2+^, Fe^3+^, Ni^2+^, Cu^2+^, Al^3+^, Mn^2+^, Ti^2+^, Pb^2+^, Zn^2+^, Cl^−^, NO_2_^−^, NO_3_^−^, SO_4_^2−^, and PO_4_^3−^ (ppm)). The second group of variables described the presence and abundance (CFU/L) of fungal genera isolated from different water and biofilm samples in different locations.

We build explainable predictive models called Predictive Clustering Trees (PCTs) that are generalised standard decision trees [28]. Models are trained on known data (annotated) and can later be used on unseen data (e.g., after retrieving additional samples) to predict the values of the output variables (predictive scenario) or to describe the data that we already have (descriptive scenario). PCTs can be parametrised to address several ML tasks. In our analysis, multi-label classification (MLC) was used. MLC is a type of multi-target prediction where input variables are used to divide the initial data in such a way that the values of the output (target) variables in the resulting clusters (leaf nodes of the PCT) are as homogeneous as possible. The resulting MLC model (a PCT) is a predictive model that is able to predict the presence of so-called labels. In our analysis, we declared specific fungi as labels, and we trained ML models to predict their presence in the water and on materials in contact with water. The PCT was generated using the CLUSPlus software package [29].

### 2.6. Model Evaluation and Parametrisation

The quality of the trained models was evaluated by calculating appropriate metrics of predictive performance. Specifically, we monitor the weighted area under the precision recall curve (AUPRC). Since we do not have a separate test set, the predictive performance was estimated with a 10-fold cross-validation procedure. PCTs were trained using all available input features and were allowed to grow until all leaf nodes contained two examples (pre-pruning).

## 3. Results

### 3.1. Location of Aquifers and Building Materials Affect the Physico-Chemical Parameters of Water

All measured physico-chemical parameters of tested drinking water samples were in accordance with the parametric values set in the European Directive 2020/2184 on the quality of water intended for human consumption (recast) [7]. Only in the sample of natural water (Ormož), elevated concentrations of Fe^3+^ (0.41 vs. parametric 0.2 ppm), Ni^2+^ (0.64 vs. parametric 0.02 ppm), Mn^2+^ (0.14 vs. parametric 0.05 ppm), and Pb^2+^ (0.07 vs. parametric 0.01 ppm) were detected. When comparing the average values between all five water plants, the differences between locations were recorded. Namely, the highest total water hardness was measured in Terbegovci (16.2 °d) and the lowest in Lukavci (8.74 °d). Terbegovci also had the highest Mg-water hardness (6.81 °d), carbonate water hardness (16.16 °d), and Mg^2+^ concentrations (29.98 ppm), while the highest values for non-carbonate water hardness were recorded in Podgrad-Segovci (3.68 °d) and Ca-water hardness in Ormož (10.52 °d). Mota had the highest values of sulphate (25.7 mg/L), and Podgrad-Segovci had the highest levels of chloride (23.8 mg/L) and nitrate (27.5 mg/L). The whole data gathered in this study are accessible through the Supplementary Material.(Supplementary Table S1).

### 3.2. Fungi Are Present in Water through the Entire Drinking Water Distribution Network 

With the isolation methods used, fungi were recovered from all water plants and water types. Overall, 78 different fungal species belonging to 51 genera were collected. Fungi were the most abundant in water from Podgrad-Segovci and the least abundant in water at Terbegovci. Abundance depended on the sampled water type and was the highest in natural, untreated water (154–766 CFU/L) and the lowest after water cleaning (4–41 CFU/L) or at the first sampling spot in households’ water network (2–64 CFU/L). Fungal numbers were elevated again with the increasing length of water networks (30–291 CFU/L) (Table 1).

**Figure 1:**
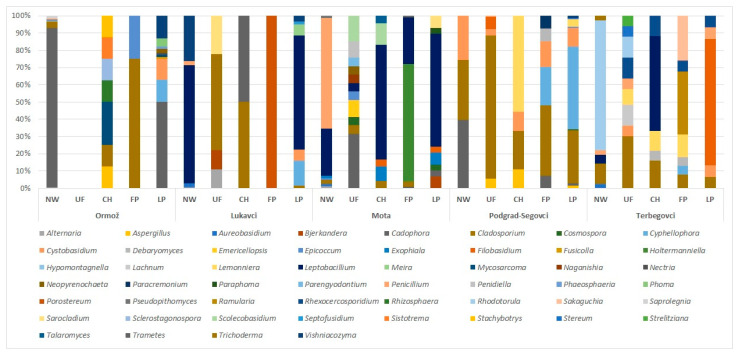
Fungal richness in different water types at five drinking water production plants. Genus *Cladosporium* was the only one detected in all five water plants. Genera *Cadophora* and *Leptobacillium* were consistently detected in water at three water plants, and *Lemonniera* at two water plants. Locally occurring genera present only at one sampling site were *Mycosarcoma* (Ormož), *Vishniacozyma* (Lukavci), *Exophiala* (Mota), *Aspergillus*, *Cystobasidium* (Podgrad-Segovci), *Penicillium*, and *Rhexocercosporidium* (Terbegovci). Water types are labeled as follows: NW—natural water; UF—water after ultrafiltration; CH—water after chlorination; FP—water at the first sampling point in the drinking water network; LP—water at the last sampling point in the drinking water network.

With 12 different presumptive species from 11 genera, fungal richness was the highest at Ormož, Terbegovci, and Podgrad-Segovci and the lowest at Lukavci (7 species, 7 genera). Species richness varied between the sampled points. It was usually the highest at the end points of the water network and the lowest immediately after chlorination or at the first point in the water network (1–5 genera) (Figure 1).

The most commonly isolated fungi from sampled natural water belonged to the genera *Cadophora*, *Cladosporium*, *Leptobacillium*, *Penicillium*, and *Rhodotorula*. The genera *Cladosporium*, *Lemonniera*, and *Leptobacillium* were the most numerous in water immediately after chlorination and at the first point in the water network. Together with *Cadophora* and *Cyphellophora,* these genera were also the most numerous in drinking water at the last sampling spots in the water network. The genus *Cladosporium* was the only genus detected at all five water plants, followed by *Cadophora* and *Leptobacillium* (3 water plants). Unidentified species of the genus *Lemonniera* were isolated from two water plants, while the genera *Mycosarcoma* (Ormož), *Vishniacozyma* (Lukavci), *Exophiala* (Mota), *Aspergillus*, *Cystobasidium* (Podgrad-Segovci), *Penicillium*, and *Rhexocercosporidium* (Terbegovci) occurred in high numbers only at one water plant but persisted throughout their water networks (Table 1, Figure 1).

Only two species of pathogenic fungi, belonging to Biosafety Level 2, were isolated. The species *Exophiala xenobiotica* was detected only in one sample of natural water and represented 1% of the isolated fungi in this sample. *Filobasidium magnum* was more common and appeared through the whole water network, with the highest numbers in natural water (up to 52 CFU/L) and at the last sampling points (22 CFU/L).

### 3.3. Water Cleaning Processes Are Effective against Fungi from Natural Water

**Figure 2:**
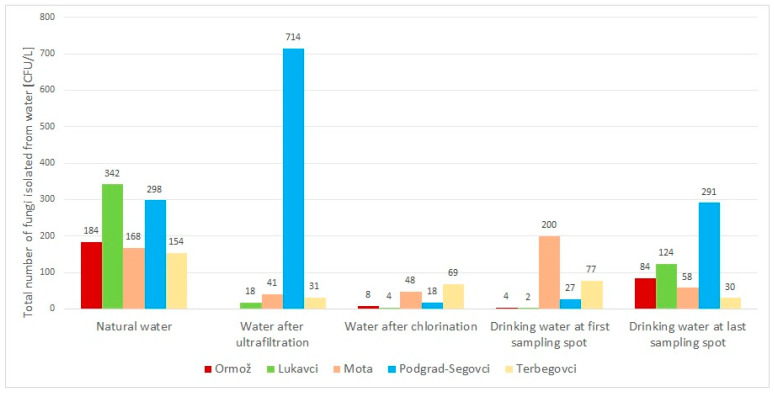
Total number of fungi isolated from different water types at five sampling locations. Natural water had, on average, the highest fungal count. The CFU/L dropped after ultrafiltration through activated carbon filters, except in Podgrad-Segovci, where ultrafiltration is performed using sand filters with a combination of UV irradiation. The effect of chlorination was reduced with the length of the water network, yielding more fungi at the last sampling spots in comparison to freshly chlorinated water.

All five water preparation plants apply water cleaning to assure the microbiological quality of the produced drinking water. Ultrafiltration with activated carbon was used in Lukavci, Mota, and Terbegovci, while a combination of sand and activated carbon was applied in Ormož. The number of isolated fungi in water after ultrafiltration with activated carbon decreased between 4.1 and 19 times in comparison to natural water. Podgrad-Segovci used filtration through sand filters in combination with UV irradiation. Here, the number of fungi increased 2.4 times in comparison to natural water (Table 1, Figure 2). Fungal species *Aureobasidium leucospermi*, *A. pullulans*, *A. subglaciale*, *Cladosporium ramotenellum*, *Exophiala xenobiotica*, *Hypomontagnella submonticulosa*, *Penicillium brevicompactum*, *P. cerradense*, *P. citrinum*, *P. kongii*, *P. rotoruae*, *Pseudopithomyces chartarum*, *Saprolegnia* sp., and *Vishniacozyma teprensis*, previously isolated from natural water, were absent after ultrafiltration (Table 1).

Purified water was additionally disinfected at all five water plants. Four (Lukavci, Mota, Podgrad-Segovci, and Terbegovci) used NaClO solutions, and one (Ormož) used chlorine (Cl_2_) gas. After chemical disinfection, the number of isolated fungi decreased in Terbegovci, Mota, Ormož, Podgrad-Segovci, and Lukavci by 55.2%, 71.4%, 95.7%, 97.5%, and 98.8%, respectively (Figure 2). Species *Alternaria* sp., *Cladosporium neolangeronii*, and *Rhodotorula* sp., present in natural water and in water after ultrafiltration, were removed with chlorination (Table 1). Fungi *Aspergillus creber*, *Cadophora malorum*, *Cladosporium allicinum*, *C. halotolerans*, *Cystobasidium slooffiae*, *Filobasidium magnum*, *Lemonniera* sp., *Leptobacillium chinense*, *Mycosarcoma maydis*, and *Rhexocercosporidium* sp. survived the chlorination process and its residual effect and were later isolated through the water distribution network (Table 1). Although chlorine reduced the number of fungi in drinking water by an average of five times, its effect decreased with exposure time and the length of the distribution network. At the last sampling spots, we detected on average two times higher numbers of fungi compared to freshly chlorinated water (Table 1, Figure 2).

### 3.4. Building Materials Selectively Promote Fungal Growth in Water Systems

**Figure 3:**
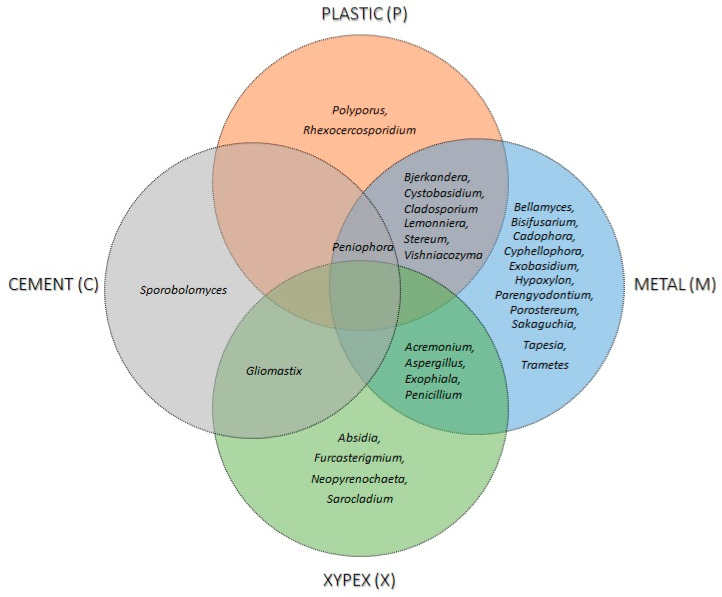
Fungi colonise different materials in contact with drinking water. The Venn diagram visualizes the intersections and co-occurrences of fungal genera isolated from different materials. Metal parts had the highest richness, with Ascomycota prevailing over Basidiomycota. The black yeasts *Cadophora*, *Cyphellophora*, and *Exophiala* colonised metal parts but were absent in samples from plastic materials. Raw cement had the lowest fungal richness, while the richness increased on cement covered with Xypex concentrate.

With cultivable methods and single barcoding identification, 32 genera and 50 presumed species of fungi colonising different materials in contact with water were identified. Out of these, 24 species (48%) were also isolated from water samples (Table 2). The highest richness was observed on materials in contact with natural water (23 species), and the lowest in contact with drinking water in cementious water storage tanks (3 species), as well as on materials in contact with freshly chlorinated water (7 species) (Table 2). Different materials were colonized by different fungi; however, sampling metals shared 7 genera with plastics and 4 with cement covered with Xypex. *Peniophora* was the only genus isolated from three different habitats (metal, plastics, and cement) (Figure 3). Among all sampled materials, metals had the highest fungal richness, with Ascomycota colonizing 59.1% of metal samples. In addition, the presence of black yeasts (*Cadophora*, *Cyphellophora*, and *Exophiala*) and other melanised fungi (*Aspergillus*, *Cladosporium*, and *Penicillium*) was detected on metals and absent from sampled plastic parts (Table 2, Figure 3). Compared to metals, 66.7% of plastics samples were colonised by a variety of Basidiomycota, while Ascomycota were less diverse.

The highest abundance was recorded on cementious material covered with Xypex concentrate (1586 CFU/cm^2^), which was 18 times higher than on cementious material without Xypex (88 CFU/cm^2^). The genus *Gliomastix* was isolated from both materials, but its numbers were 246 times higher on Xypex than on raw cement. The abundance was also high on a rubber sample in contact with natural water (74 CFU/cm^2^). The lowest abundance was recorded for metal ($\bar{x}$ = 8.7 CFU/cm^2^ per sample) and plastic materials ($\bar{x}$ = 2.5 CFU/cm^2^ per sample).

Among isolates from materials in contact with drinking water, only two presumptive species, *Exophiala xenobiotica* and *Sarocladium kiliense* (1 and 7 CFU/cm^2^, respectively), were categorized as Biosafety-2 fungi. None of them was isolated from drinking water at the consumers’ taps.

### 3.5. Presence of Fungi in Water and on Materials Depends on the Location and Water Type

**Figure 4:**
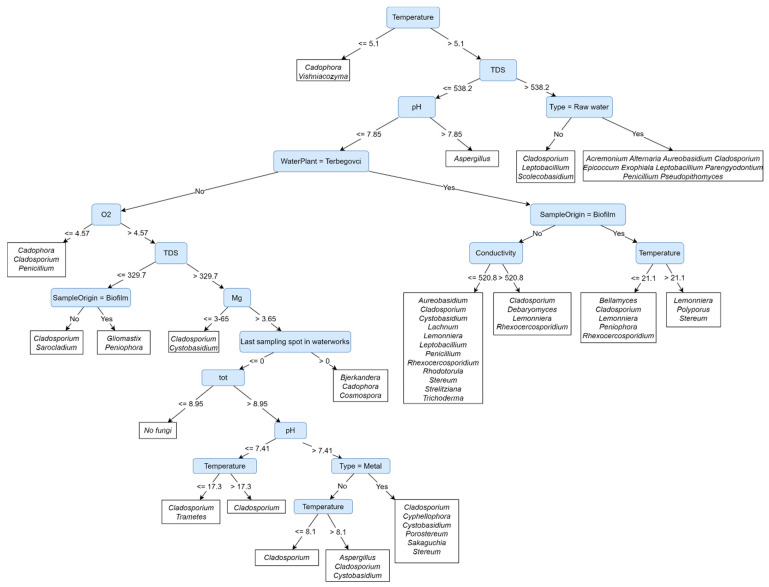
A multi—label classification Predictive Clustering Tree (PCT) that predicts fungi (labels) based on environmental factors. A prediction for a sample is made by traversing the tree and comparing the values of the samples’ attributes to the conditions in the nodes in the tree. In this case, *Temperature* was determined as the most important attribute, therefore, one should compare the samples’ value of the *Temperature* attribute and traverse the tree to the right when temperature is higher than 5.1 °C or left otherwise. At some point, the traversal ends in a terminal (leaf) node of the tree, which holds the predictions of fungi.

The structure of the Predictive Clustering Tree (PCT, Figure 4) reveals the most influential factors for fungal presence in water and on materials in contact with water. Figure 4 depicts a model that makes predictions about fungal communities based on environmental factors. The first and most important factor is water temperature, predicting the majority of fungi when >5.1 °C. The highest richness is predicted to be reached in water and biofilm samples with a water temperature > 5.1 °C, under cascades of factors describing natural water and biofilms in Mota, Terbegovci, and Podgrad-Segovci. The lowest richness with only one or two genera is predicted for water and biofilm samples after chlorination and at the first sampling spot in drinking water distribution networks, regardless of the location. Fungal presence can be divided into three major groups: ubiquitous fungi, locally occurring fungi, and fungi affected by water cleaning processes.

The genera *Cadophora*, *Cladosporium*, *Cystobasidium*, and *Stereum* are ubiquitous fungi appearing in more than one leaf. They are predicted to be found regardless of the sampling location and sample type (water or biofilm). However, *Cladosporium*, *Cystobasidium*, and *Stereum* were absent from a sample with a water temperature ≤ 5.1 °C (Figure 4). On the other hand, the genera *Lemonniera*, *Rhexocercosporidium*, and *Leptobacillium* are predicted to be isolated from samples with a water temperature > 5.1 °C under the cascades of environmental factors strictly related to the location of the main water source (Terbegovci and Mota). There, they can be isolated regardless of the types and materials in contact with water (Figure 4). The third group of fungi is related to water type. All have been predicted to be present in samples with a water temperature > 5.1 °C. *Aureobasidium* and *Penicillium* have been predicted under the cascades of environmental factors describing natural water and materials in contact with this water in Mota, Terbegovci, Podgrad-Segovci, and Ormož. The genus *Aspergillus* is predicted to be isolated from chlorinated water or materials in contact with such water. The main water source in all cases of *Aspergillus* prediction is artificially enriched groundwater with TDS ≤ 538 ppm (Ormož, Podgrad-Segovci) (Figure 4).

## 4. Discussion

The surface area of Slovenia is dominated by sedimentary rocks and deposits ideal for the formation of groundwater aquifers, and most households can use underground water as their main water source [30]. A special feature in the south and south-east parts of the country are the karst aquifers, which are more prone to anthropogenic pollution due to their calcareous structure and thinner layers [4]. Basins and valleys in central Slovenia and the north-east part of the country are also points at risk for water contamination. Reasons for this are the growing urbanization, industrialization, and extensive agriculture seen in other developed countries [31]. These factors, together with geological features and natural water sources, affect the physico-chemical composition of drinking water [2]. Microorganisms, in particular bacteria, in oligotrophic drinking water systems are rarely present in excessive numbers since they are strictly regulated. On the other hand, microorganisms colonising materials are not officially regulated, although Slovenia in 2016 issued guidelines for materials in contact with drinking water [32]. Fungi are not specifically monitored, although their presence was discussed in relation to novel, cementious drinking water storage tanks [17]. Following the recommendations in the research of Novak Babič et al. [2] and the EU EMEG group [8], we used a culturable approach to investigate the effect of location, natural water source, different water cleaning methods, and the choice of material on the presence and abundance of fungi in drinking water. Our study focused on culturable mycobiota in closely located water plants (~45 km distance) between two large rivers, the Drava and Mura.

### 4.1. Aquifer Location and Natural Water Catchment Methods Have a Crucial Effect on Mycobiota in the Drinking Water Distribution System

One of the main findings related the presence and abundance of locally dominant genera to the location of aquifers and catchment methods for natural water. Physico-chemical factors likely differed due to geological differences, the presence of mineral water layers, and groundwater capture methods [33]. The water plant Terbegovci uses groundwater, which is located near mineral water layers and is harder in comparison to other samples, with a higher pH, carbonate, magnesium, and total water hardness [34]. On the other hand, Podgrad-Segovci provides artificially made groundwater, with the Mura seeping through infiltration fields. The effect of surface water surrounded by agricultural areas is seen through higher concentrations of chloride and nitrate [35]. These factors particularly affect the presence of locally occurring genera, such as *Lemonniera*, *Rhexocercosporidium*, *Leptobacillium*, and *Filobasidium*, with most isolates belonging to new, undescribed species. Locally significant genera were isolated from one or two water preparation plants only but were consistently present throughout the whole water network regardless of water disinfection. A similar effect was discussed in previous studies [2,33,36] but was so far not documented inside one country within such short distances (Ormož—Podgrad-Segovci ~45 km). The PCT (Figure 4) also reveals the effect of location on fungal biota, where water temperature, TDS, and water type are identified as the most influential factors for fungal presence in water. The main reason for differences in water temperature is the time of sampling. Water in Ormož was taken in January, while the rest of the sampling was carried out in April and May. The cascades of these factors lead to leaves in the decision tree, grouping fungi almost strictly according to the location of water preparation plants and consequently the location of natural water (Figure 4).

The availability and access of natural water collided with the choice of water catchment methods [31], which seem to have the biggest effect on the mycobiota of drinking water [33]. Differences between true groundwater and river-derived water were seen through the presence of fungi either related to groundwater and polar-alpine habitats on one side and plant- or soil-related genera on the other side. In our study, Ormož and Podgrad-Segovci both use artificially made groundwater prepared from nearby rivers Drava and Mura. Despite slightly different catchment methods between these water preparation plants, the higher richness of fungi from the genera *Aspergillus*, *Aureobasidium*, *Cadophora*, *Cladosporium*, *Filobasidium*, and *Penicillium*, known to be isolated from surface water and commonly also from plants, their roots, and soil, were isolated in both cases [37,38,39]. On the other hand, Lukavci, Mota, and Terbegovci use deep wells with direct access to the groundwater. From these samples, numerous fungi of the genera *Exophiala*, *Cyphellophora*, and *Cystobasidium* previously recorded from other European groundwater sources, rivers, and glaciers were isolated [40,41,42,43,44]. Mycobiota differed in groundwater from Terbegovci, probably due to the borders with mineral water layers. Many strains of potentially new species have been isolated at this location. They are taxonomically closest to the genera *Lemonniera*, *Rhexocercosporidium*, and *Rhodotorula*. Among these, *Rhodotorula* was already associated with mineral water [45].

### 4.2. Chlorine-Based Disinfection with Residual Effect over Time and Length Lowers Fungal Abundance and Richness

Data from 2017 reported 866 active water supplies in Slovenia, of which 31% do not require chemical disinfection, 9% use it occasionally, and 60% use permanent disinfection [5]. The first choice is chlorine and its compounds. UV radiation is becoming increasingly used as well [5,46]. In our study, the number of fungi decreased 4.1–19 times after ultrafiltration with activated carbon, which is in accordance with previous reports where ultrafiltration removed up to 99.9% of microorganisms [46]. At this stage of water preparation, only in Podgrad-Segovci did the fungal numbers increase in comparison to natural water. Here, combined filtration with sand and UV treatment is used instead of activated carbon. The increased numbers are likely the consequence of filter saturation [47]. In the disinfection step, only Ormož used chlorine gas (Cl_2_), while others used NaOCl. Both have been effective against fungi, decreasing their numbers by 55–98.8%, which is in accordance with the reports on chlorination removing up to 99.99% of microorganisms [36,46,48]. The chlorination effect also depends on the concentration of dissolved organic and inorganic materials and water hardness [49] and was thus the least effective in Terbegovci and Mota, with the highest total water hardness (both > 16 °d). A variety of fungi can survive the disinfection of water (Table 1, Figure 4), and this represents an inoculum entering drinking water networks [50]. Our findings also confirmed the fading effect of residual chlorine with increasing fungal numbers after the prolonged time and length of the water network in all five studied water networks [51].

### 4.3. The Choice of Building Materials Makes Selective Pressure on Water-Borne Fungi

Fungi may colonise diverse spectrum of materials and form biofilm regardless of the hydrophobicity or hydrophilicity of the material [50]. In vitro studies have shown such ability for the genera *Aspergillus*, *Aureobasidium*, *Candida*, *Naganishia*, *Penicillium*, *Saccharomyces*, and *Trichoderma* [12,52]. In addition to these, biofilms from the water systems of private homes, hospitals, and industrial plants contained fungi of the genera *Cladosporium*, *Exophiala*, *Fusarium*, *Malassezia*, *Scolecobasidium* (previously *Ochroconis*), *Phialophora*, *Phoma*, *Rhinocladiella*, and *Rhodotorula* [2]. However, our results not only describe fungi colonizing materials but also show differences in fungal biota on materials in relation to the type of material and type of water in contact with the material. Forty-eight percent of fungi found at certain locations and in certain water types have also been isolated from the material in contact with this water (Table 2, Figure 4). Other species may have a seasonal water-borne origin but could as well be associated with the surrounding environment, such as soil, plants, animals, humans, and air. As in the case of water, the decrease in fungal abundance on materials in contact with water was recorded after water cleaning procedures [53].

The occurrence of fungi in water networks was seen due to the choice of material, as discussed in Douterelo et al. (2020) [54]. Rough surfaces, such as cement, silicones, and rubbers, are more prone to colonization with microbes than smoother plastics or metals [53]. Biofilms on such materials have longer durability, and the colonized materials degrade faster, which may affect the quality of the material and water [17,18]. Such a phenomenon was also recorded in our study, where elevated concentrations of Fe^3+^, Mn^2+^, and Pb^2+^ in the sample of natural water in Ormož and Ni^2+^ at the last sampling point were detected. While the presence of Fe^3+^ and Mn^2+^ could be linked to the features of natural water before aeration [55], the presence of Pb^2+^ and Ni^2+^ indicates metals’ leaking [32]. The fungal abundance here was 1.6 times higher in comparison to the same samples in other water plants.

The difference among materials in contact with the same type of water was expressed even more in cement in comparison to cement covered with Xypex. Fungi were 3.8 times more abundant on Xypex-covered cement than on raw cement, with the species *Furcasterigmium furcatum*, *Gliomastix murorum*, *Neopyrenochaeta* sp., and *Sarocladium kiliense* being the most common. Xypex coating particularly affected the abundance of *G. murorum*, with it being 246 times more numerous in comparison to rough cement surfaces. Consequently, the fungal abundance in water in contact with the coating was two-times higher than in water samples that were in contact with rough cement. The results are in accordance with the previous study on water of karstic origin, where the fungal abundance of *Furcasterigmium*, *Gliomastix*, and *Gloeotinia* on surfaces with Xypex concentrate exceeded the number on other materials by a factor of 6.5 [17].

Similar to cement and its coating, *Acremonium*, *Aspergillus*, *Cladosporium*, and *Penicillium* occurred more often on the rough surface of a rubber pipe in comparison to the metal pipe to which the rubber pipe was attached. On the contrary, *Cadophora* was more abundant on the metal parts and less on the rubber parts of the pipe. Such results were expected, as it was already recorded in previous studies that spores of the above-mentioned genera attach more easily to the rough rubber than to the smooth metal [2,53].

In general, metal pipes had the lowest recorded abundance of fungi but expressed the highest richness. Similarity was observed by Douterelo et al. (2020) [54], where cast-iron pipes promoted the most stable microbial networks with the highest fungal richness. In our study, ascomycetous melanised fungi from the genera *Aspergillus*, *Cadophora*, *Cladosporium*, *Cyphellophora*, and *Exophiala* dominated on metals in contact with water. This phenomenon was reported earlier, where melanin was described in association with the metal-binding of various substrates [56]. While present on 59.1% of metal samples, Ascomycota were much less abundant and diverse on plastics, where they were isolated from only 33.3% of the samples. In contrast, with 66.7% of positive samples, Basidiomycota dominated plastic materials. These included the genera *Cystobasidium*, *Vishniacozyma*, *Bjerkandera*, and *Stereum*. Many studies report fungi from plastic materials, but contrary to our results, higher numbers of Ascomycota are usually reported [57].

### 4.4. Health Risk Due to Fungi in Water Networks

People are exposed to fungi from water through drinking, showering, cooking, and recreational activities directly through their skin and inhalation of aqueous aerosols [33]. In the case of fungal infections in people with immune deficiency, the increased occurrence of antimycotic resistance in the genera *Aspergillus*, *Candida*, *Fusarium*, *Mucor*, and black yeasts is of the greatest concern, according to the latest list of emerging fungi issued by WHO [58]. Although thermotolerant *Aspergillus* spp. and *Candida parapsilosis* have been isolated from water in Europe [2] as well as in previous Slovenian studies [17,19], they were not isolated in any of the samples in this study. Among isolates, only the presumptive species *Exophiala xenobiotica*, *Filobasidium magnum*, and *Sarocladium killiense* belonged to Biosafety Level 2 (Table 1 and Table 2) [59]. All of these appeared in the water distribution systems in low numbers in water and on materials and do not present a health risk. However, many fungal strains that are not yet taxonomically described were isolated and also have no known effect on human health. Any elevated numbers of these fungi should thus be taken into consideration in cases of health-related outbreaks or association with mycotoxins intoxication [60].

## 5. Conclusions

Our study followed the richness and abundance of fungi through the large drinking water networks of five water preparation plants. They acquire water from different aquifers, use different cleaning methods, and use different materials for water transport. Out of these, the aquifer location and the type of natural water were the most important factors affecting the mycobiota of drinking water. Natural and drinking water represent a source of transmission for many new, undescribed species. While certain genera are strictly connected to the main water source location, fungi could serve as an important future indicator following environmental changes and their effects on the quality of natural water sources. Cleaning processes reduce fungal abundance in drinking water; however, some species survive disinfection and are present in low numbers throughout the drinking water distribution systems. Due to the decrease in chlorine residual effect with length and time, the number of surviving fungi increased at the last sampling spots. Although they are mainly non-pathogenic, attention is needed in cases of sudden increases in fungi in drinking water. Fungi present in water were also present on materials in contact with water. The results show a huge increase in the richness and abundance of fungi on porous materials with a rough surface in comparison to materials with smooth surfaces, such as metals and plastics. Caution is recommended when using porous materials, as they positively affected the colonization and growth of fungi, which consequently increased their numbers in the water. An interesting phenomenon was recorded as more ascomycetous: melanized fungi were isolated from metals in comparison to plastics. Plastic, on the other hand, was preferred by fungi from the Basidiomycota kingdom. This suggests the selective nature of certain fungal groups depending on the use of materials in water networks. Although more studies on the phenomenon are needed, the use of fungal target groups in guidelines evaluating the biodegradation of certain materials in drinking water networks is recommended. We used a cultivable approach and single barcoding molecular markers for the identification of strains by genus and species levels. To confirm the taxonomical classification of the listed species, additional molecular barcodes should be used.

## Figures and Tables

**Table 1:** Richness and abundance of fungi from different water types at five drinking water production plants.

Fungal Species According to a Single Barcode Marker	CFU/L ^1^ in Different Types of Water								EXF ^2^ No.	GenBank ^3^ No.
	IF	NW	UF	CH	FP	WS-C	WS-X	LP		
*Alternaria* sp.		2	2						16274, 16619, 16574, 16593	OP675912, OP675913, OP675914, OP675915 (ITS)
*Aspergillus creber*			32	≤2				4	16276, 16759, 16938, 16773	OP700416, OP700417, OP700418, OP700419 (*benA*)
*Aspergillus westerdijkiae*			8						16761	OP700424 (*benA*)
*Aureobasidium leucospermi*	112								16809	OP675916 (ITS)
*Aureobasidium pullulans*		≤4							16594, 16781	OP675918, OP675917 (ITS)
*Aureobasidium subglaciale*		8							16793	OP675919 (ITS)
*Bjerkandera adusta*			2					4	16576, 16647	OP675921 (ITS), OP675923 (ITS)
*Cadophora malorum*		≤170	13		2			≤42	16268, 16283, 16643, 16646, 16811,16947	OP675931, OP675934, OP675938, OP675941, OP675942, OP675943 (ITS)
*Cadophora sabaouae*					2				16940	OP675945 (ITS)
*Cadophora* sp.								2	16618	OP675946 (ITS)
*Cladosporium allicinum*		4	≤6	6	≤6	2		2	16271, 16573, 16580, 16578, 16598, 16645, 16825, 16787	OP653735, OP653736, OP653738, OP653737, OP653739, OP653740, OP653742, OP653743 (*act*)
*Cladosporium halotolerans*	652	82	≤592	≤4	11		2	≤90	16275, 16620, 16630, 16819, 16790, 16752, 16755, 16760, 16763, 16767, 16771	OP653746, OP653748, OP653749, OP653763, OP653764, OP653753, OP653756, OP653757, OP653758, OP653761, OP653762 (*act*)
*Cladosporium neolangeronii*		22	2						16794, 16936	OP653765, OP653768 (*act*)
*Cladosporium proteacearum*			2						16572	OP653769 (*act*)
*Cladosporium pseudocladosporioides*		≤4		≤5	3				16270, 16590, 16605, 16824, 16789	OP653770, OP653771, OP653772, OP653774, OP653775 (*act*)
*Cladosporium ramotenellum*		18							16777	OP653776 (*act*)
*Cladosporium* sp.					3				16281	OP653777 (*act*)
*Cosmospora* sp.								2	16649, 16945	OP675948, OP675950 (ITS)
*Cosmospora viridescens*			2						16601	OP675947 (ITS)
*Cyphellophora reptans*					≤6			24	16963, 16941, 16943	OP675956, OP675954, OP675955 (ITS)
*Cyphellophora sessilis*						8		≤103	16387, 16795, 16636, 16814	OP675957, OP675960, OP675959, OP675961 (ITS)
*Cyphellophora* sp.								12	16944	OP675962 (ITS)
*Cystobasidium lysinophilum*								5	16285	OP642011 (LSU)
*Cystobasidium slooffiae*		≤76	2	2	4			≤32	16284, 16634, 16959, 16830, 16756, 16757, 16812, 16813, 16772	OP642014, OP642013, OP642021, OP642022, OP642016, OP642017, OP642018, OP642019, OP642020 (LSU)
*Debaryomyces hansenii*				4	≤4				16822, 16827, 16765	OP675964, OP675965, OP675963 (ITS)
*Emericellopsis* sp.			4						16599	OP675966 (ITS)
*Epicoccum* sp.		2	2		1			2	16280, 16596, 16611, 16770	OP675968, OP675969, OP675970, OP675971 (ITS)
*Exophiala angulospora*				2					16799	OP675974 (ITS)
*Exophiala cancerae*				2					16609	OP675975 (ITS)
*Exophiala equina*								4	16650	OP675976 (ITS)
***Exophiala xenobiotica***		**2**							**16595**	**OP675978 (ITS)**
***Filobasidium magnum***			**52**	**2**				**≤22**	**16608, 16616, 16791, 16758**	**OP675979, OP675980, OP675982, OP675981 (ITS)**
*Fusicolla ossicola*								1	16287	OP675984 (ITS)
*Holtermanniella takashimae*					136				16612	OP642023 (LSU)
*Hypomontagnella submonticulosa*		1							16269	OP675985 (ITS)
*Lachnum virgineum*			4						16957	OP675987 (ITS)
*Lemonniera* sp.			3	≤10	10			12	16955, 16960, 16962, 16937, 16942	OP675996, OP675998, OP675999, OP675990, OP675992 (ITS)
*Leptobacillium chinense*		≤234	2	≤38	54		30	≤82	16571, 16582, 16577, 16589, 16597, 16606, 16613, 16615, 16780, 16785	OP676001, OP676003, OP676002, OP676004, OP676005, OP676006, OP676007, OP676008, OP676009, OP676010 (ITS)
*Meira* sp.								8	16633	OP676011 (ITS)
*Mycosarcoma maydis*		1		2				1	16273, 16279, 16286	OP676012, OP676013, OP676014 (ITS)
*Naganishia cerealis*			2						16603	OP676015 (ITS)
*Nectria flavoviridis*								1	16289	OP676016 (ITS)
*Neopyrenochaeta* sp.			2					2	16302, 16640	OP676017, OP676019 (ITS)
*Paracremonium* sp.					2				16768	OP676020 (ITS)
*Paraphoma radicina*								2	16648	OP676021 (ITS)
*Parengyodontium torokii*			2						16602	OP676023 (ITS)
*Penicillium bialowiezense*								2	16965	OP700425 (*benA*)
*Penicillium brevicompactum*		96							16588	OP700426 (*benA*)
*Penicillium cerradense*		≤5							16587, 16782	OP700429, OP700430 (*benA*)
*Penicillium citrinum*		1							16272	OP700431 (*benA*)
*Penicillium kongii*	2	1							16591, 16810	OP700432, OP700433 (*benA*)
*Penicillium rotoruae*		8							16570	OP700437 (*benA*)
*Penicillium rubens*			≤4						16783, 16762	OP700440, OP700439 (*benA*)
*Penidiella* sp.			4			9	11		16796, 16797, 16642	OP676024, OP676025, OP676026 (ITS)
*Phaeosphaeria* sp.								1	16290	OP676031 (ITS)
*Phoma herbarum*								4	16288	OP676032 (ITS)
*Porostereum spadiceum*					2				16631	OP676034 (ITS)
*Pseudopithomyces chartarum*		2							16592	OP676036 (ITS)
*Ramularia lethalis*					28				16961	OP676037 (ITS)
*Rhexocercosporidium* sp.			4	8	5			2	16821, 16823, 16826, 16829	OP676040, OP676041, OP676042, OP676044 (ITS)
*Rhizosphaera macrospora*				1					16277	OP676045 (ITS)
*Rhodotorula* sp.		116	4						16778, 16784	OP642024, OP642025 (LSU)
*Sakaguchia* sp.					20				16788	OP676047 (ITS)
*Saprolegnia* sp.		3							16267	OP676048 (ITS)
*Sarocladium implicatum*			4			2	4		16575, 16575, 16583	OP676049, OP676049, OP676051 (ITS)
*Sarocladium strictum*								4	16617	OP676053 (ITS)
*Sclerostagonospora cycadis*				1					16278	OP676054 (ITS)
*Scolecobasidium* sp.			6	6		4			16581, 16600, 16610	OP676055, OP676056, OP676058 (ITS)
*Septofusidium berolinense*								2	16635	OP676059 (ITS)
*Sistotrema* sp.				1					16382	OP676060 (ITS)
*Stachybotrys chartarum*				1					16383	OP676061 (ITS)
*Stereum* sp.			2					2	16820, 16774	OP676066, OP676063 (ITS)
*Strelitziana* sp.			2						16956	OP676067 (ITS)
*Talaromyces amestolkiae*				2					16607	OP700443 (*benA*)
*Trametes versicolor*				2	2				16621, 16614	OP676069, OP676070 (ITS)
*Vishniacozyma carnescens*								≤11	16282, 16769	OP642029, OP642028 (LSU)
*Vishniacozyma heimaeyensis*								4	16637	OP642030 (LSU)
*Vishniacozyma tephrensis*		90							16632	OP642031 (LSU)

Legend: ^1^ CFU/L—Colony Forming Unit; number of units forming colonies in one liter of water. ^2^ EXF No.—Number of fungal strains deposited in the Culture Collection of Extremophiles (Ex), Infrastructural Center Mycosmo, Biotechnical Faculty, University of Ljubljana, Slovenia. ^3^ GenBank No.—Number assigned to sequences deposited in the international database of the National Center for Biotechnology Information, Bethesda, USA. IF—Water from the infiltration field; NW—Natural water; UF—Water after ultrafiltration; CH—Water after chlorination; FP—Water at the first sampling point in the drinking water network; WS-C—Water from a water storage tank made of cement; WS-X—Water from a water storage tank coated with Xypex; LP—Water at the last sampling point in the drinking water network. Bold—presumptive fungal species belonging to Biosafety Level 2.

**Table 2:** Richness and abundance of fungi from different materials in contact with water at five drinking water production plants.

Fungal Species According to a Single Barcode Marker	CFU/cm^2^ on Different Materials in Contact with Water ^1^						EXF ^2^ No.	GenBank ^3^ No.
	NW	CH	FP	WS-C	WS-X	LP		
*Absidia glauca*					X: 1		16263	OP675900 (ITS)
*Acremonium sclerotigenum*	R: 13				X: 416		16252, 16264	OP675904, OP675909 (ITS)
*Acremonium* sp.	M: 3						16798	OP675911 (ITS)
*Aspergillus creber* *	R: 2				X: 505		16251, 16262	OP700414, OP700415 (*benA*)
*Aspergillus protuberus*		M: 1					16259	OP700421 (*benA*)
*Aspergillus puulaauensis*					X: 95		16261	OP700422 (*benA*)
*Aspergillus* sp.		M: 1					16373	OP700423 (*benA*)
*Bellamyces quercus*			M: 1				16952	OP675920 (ITS)
*Bisifusarium dimerum*						M: 60	16566	OP653779 (*tef1*)
*Bjerkandera adusta* *			P: 1			M: 1	16586, 16751	OP675922, OP675924 (ITS)
*Cadophora malorum* *	R: 1, M: 9					M: 1	16256, 16301, 16378	OP675926, OP675925, OP675930 (ITS)
*Cladosporium allicinum* *	R: 4						16254	OP653734 (*act*)
*Cladosporium anthropophilum*			P: 1				16266	OP653744 (*act*)
*Cladosporium halotolerans* *	M: ≤2	M: 1					16255, 16563, 16638, 16747, 16749	OP653745, OP653747, OP653750, OP653751, OP653752 (*act*)
*Cladosporium neolangeronii* *	M: 1	M: 1					16804, 16806	OP653766, OP653767 (*act*)
*Cladosporium pseudocladosporioides* *	P: 1						16775	OP653773 (*act*)
*Cladosporium westerdijkiae*	R: 1						16250	OP653778 (*act*)
*Colacogloea* sp.	R: 1						16366	OP642010 (LSU)
*Cyphellophora reptans* *	M: 1						16626, 16805	OP675951, OP675952 (ITS)
*Cyphellophora sessilis* *	M: 1						16931	OP675958 (ITS)
*Cystobasidium lysinophilum*			P: 1				16584	OP642012 (LSU)
*Cystobasidium slooffiae* *	M: 1						16933	OP642015 (LSU)
*Epicoccum* sp. *	R: 1						16253	OP675967 (ITS)
*Exobasidium warmingii*	M: 4						16792	OP675972 (ITS)
*Exophiala angulospora* *					X: 22		16624	OP675973 (ITS)
***Exophiala xenobiotica*** *						**M: 1**	**16379**	**OP675977 (ITS)**
*Furcasterigmium furcatum*					X: 49		16568	OP675983 (ITS)
*Gliomastix murorum*				C: 2	X: 492		16628, 16567	OP675903, OP675901 (ITS)
*Hypoxylon howeanum*			M: 1				16565	OP675986 (ITS)
*Lemonniera* sp. *			M: 1			P: 1	16950, 16953, 16935	OP675993, OP675995, OP675989 (ITS)
*Neopyrenochaeta* sp. *					X: 1		16623	OP676018 (ITS)
*Parengyodontium* sp. *	M: 5						16639	OP676022 (ITS)
*Penicillium buchwaldii*	M: 1						16258	OP700427 (*benA*)
*Penicillium cerradense* *	R: 5						16248	OP700428 (*benA*)
*Penicillium pancosmium*	R: 7						16368	OP700434 (*benA*)
*Penicillium roseopurpureum*	R: 32 M: 1						16257, 16367	OP700436, OP700435 (*benA*)
*Penicillium rubens* *					X: 3		16260	OP700438 (*benA*)
*Penicillium sanguifluum*	R: 7						16249	OP700441 (*benA*)
*Peniophora quercina*	M: 1, P: 1		M: 1	C: 4			16625, 16629, 16816, 16817	OP676027, OP676028, OP676029, OP676030 (ITS)
*Polyporus lepideus*						P: 1	16954	OP676033 (ITS)
*Porostereum spadiceum* *		M: 1					16748	OP676035 (ITS)
*Rhexocercosporidium* sp. *	P: 1						16948	OP676038 (ITS)
*Sakaguchia dacryoidea* *		M: 3					16750	OP676046 (ITS)
***Sarocladium kiliense***					**X: 7**		**16622**	**OP676052 (ITS)**
*Sporobolomyces ruberrimus*				C: 82			16627	OP642026 (LSU)
*Stereum* sp. *		M: 1				P: 1	16776, 16818, 16807	OP676064, OP676065, OP676062 (ITS)
*Tapesia fusca*			M: 1				16564	OP676068 (ITS)
*Trametes versicolor* *			M: 1				16808	OP676071 (ITS)
*Vishniacozyma carnescens* *			P: 1				16585	OP642027 (LSU)
*Vishniacozyma victoriae*						M: 1	16377	OP642032 (LSU)

Legend: ^1^ CFU/cm^2^—Colony Forming Unit; number of units forming colonies on the surface of 1 cm^2^. ^2^ EXF No.—Number of fungal strains deposited in the Culture Collection of Extremophiles (Ex), Infrastructural Center Mycosmo, Biotechnical Faculty, University of Ljubljana, Slovenia. ^3^ GenBank No.—Number assigned to sequences deposited in the international database of the National Center for Biotechnology Information, Bethesda, USA. *—Fungi, isolated from both habitats, water, and materials in contact with water. NW—Natural water; CH—Water after chlorination; FP—Water at the first sampling point in the drinking water network; WS-C—Water from a water storage tank made of cement; WS-X—Water from a water storage tank coated with Xypex; LP—Water at the last sampling point in the drinking water network; M—Metal; R—Rubber; P—Plastic; C—Cement; X—Cementious coating, Xypex. Bold—presumptive fungal species belonging to Biosafety Level-2.

## Data Availability

Data sharing is not applicable to this article.

## Supplementary Materials

The following supporting information can be downloaded at: https://www.mdpi.com/article/10.3390/jof9111086/s1, Supplementary Table S1: Physico-chemical parameters of water samples.

## References

[1] UNDP (2018). Nature for Water, Nature for Life: Nature-Based Solutions for Achieving the Global Goals.

[2] Novak Babič M., Gunde-Cimerman N., Vargha M., Tischner Z., Magyar D., Veríssimo C., Sabino R., Viegas C., Meyer W., Brandão J. (2017). Fungal Contaminants in DrinkingWater Regulation?. A Tale of Ecology, Exposure, Purification and Clinical Relevance. Int. J. Environ. Res. Public Health.

[3] Janža M., Meglič P., Šram D., Adrinek S., Koren K. (2021). Možnosti za Povečanje Potenciala Lokacij za Akvakulturo na Celinskih Podzemnih Vodah v Republiki Sloveniji.

[4] Vlada Republike Slovenije (2016). Načrt Upravljanja Voda na Vodnem Območju Donave za Obdobje 2016–2021.

[5] NLZOH (2017). Monitoring Pitne Vode 2017—Letno Poročilo o Kakovosti Pitne Vode v Letu 2017.

[6] Percival L.S., Yates V.M., Williams W.D., Chalmers R.M., Gray F.N. (2014). Microbiology of Waterborne Diseases.

[7] EEC (2020). Directive (EU) 2020/2184 of the European Parliament and of the Council of 16 December 2020 on the quality of water intended for human consumption (recast). Off. J. Eur. Union.

[8] Niegowska M.Z., Pitkänen T., Sommer R., Brandão J., Bonadonna L., Budišová D., Burlion N., Gassilloud B., Pissarides N., Proksova M. (2022). Recast Drinking Water Directive—State of Play: Guidance Note for the Analysis of Microbiological Parameters.

[9] Alonso R., Pisa D., Fernandez-Fernandez A.M., Rabano A., Carrasco L. (2017). Fungal infection in neural tissue of patients with amyotrophic lateral sclerosis. Neurobiol. Dis..

[10] French P.W., Ludowyke R.I., Guillemin G.J. (2019). Fungal-contaminated grass and well water and sporadic amyotrophic lateral sclerosis. Neural Regen. Res..

[11] Afonso T.B., Simões L.C., Lima N. (2021). Occurrence of filamentous fungi in drinking water: Their role on fungal-bacterial biofilm formation. Res. Microbiol..

[12] Siqueira V.M., Oliveira H.M.B., Santos C., Paterson R.R.M., Gusmão N.B., Lima N. (2011). Filamentous Fungi in drinking water, particularly in relation to biofilm formation. Int. J. Environ. Res. Public Health.

[13] Kadaifciler D.G., Demirel R. (2018). Fungal contaminants in man-made water systems connected to municipal water. J. Water Health.

[14] Moat J., Rizoulis A., Fox G., Upton M. (2016). Domestic shower hose biofilms contain fungal species capable of causing opportunistic infection. J. Water Res..

[15] Andersen B., Frisvad J.C., Søndergaard I., Rasmussen I.S., Larsen L.S. (2011). Associations between fungal species and water-damaged building materials. Appl. Environ. Microbiol..

[16] Del Olmo G., Husband S., Sánchez Briones C., Soriano A., Calero Preciado C., Macian J., Douterelo I. (2021). The microbial ecology of a Mediterranean chlorinated drinking water distribution systems in the city of Valencia (Spain). Sci. Total Environ..

[17] Novak Babič M., Gunde-Cimerman N. (2021). Water-Transmitted Fungi Are Involved in Degradation of Concrete Drinking Water Storage Tanks. Microorganisms.

[18] Bertron A. (2014). Understanding interactions between cementitious materials and microorganisms: A key to sustainable and safe concrete structures in various contexts. Mater. Struct..

[19] Novak Babič M., Zalar P., Ženko B., Džeroski S., Gunde-Cimerman N. (2016). Yeasts and yeast-like fungi in tap water and groundwater, and their transmission to household appliances. Fungal Ecol..

[20] Van den Ende A.H.G., de Hoog G.S. (1999). Variability and molecular diagnostics of the neurotropic species Cladophialophora bantiana. Stud. Mycol..

[21] White T.J., Bruns T., Lee S., Taylor J., Innis M.A., Gelfand D.H., Sninsky J.J., White T.J. (1990). Amplification and direct sequencing of fungal ribosomal RNA genes for phylogenetics. PCR Protocols: A Guide to Methods and Applications.

[22] Boekhout T., Kurtzman C.P., Wolf K. (1996). Principles and methods used in yeast classification, and an overview of currently accepted yeast genera. Nonconventional Yeasts in Biotechnology.

[23] Carbone I., Kohn L.M. (1999). A method for designing primer sets for speciation studies in filamentous ascomycetes. Mycologia.

[24] Glass N., Donaldson G. (1995). Development of primer sets designed for use with the PCR to amplify conserved genes from filamentous ascomycetes. Appl. Environ. Microbiol..

[25] O’Donnell K., Kistler H.C., Cigelnik E., Ploetz R.C. (1998). Multiple evolutionary origins of the fungus causing Panama disease of banana: Concordant evidence from nuclear and mitochondrial gene genealogies. Proc. Natl. Acad. Sci. USA.

[26] Kumar S., Stecher G., Tamura K. (2016). MEGA7: Molecular Evolutionary Genetics Analysis Version 7.0 for Bigger Datasets. Mol. Biol. Evol..

[27] Altschul S.F., Gish W., Miller W., Myers E.W., Lipman D.J. (1990). Basic local alignment search tool. J. Mol. Biol..

[28] Blockeel H., De Raedt L. (1998). Top-down induction of first-order logical decision trees. Artif. Intell..

[29] Petković M., Levatić J., Kocev D., Breskvar M., Džeroski S. (2023). CLUSplus: A decision tree-based framework for predicting structured outputs. SoftwareX.

[30] Mikulič Z., ARSO (2009). Symposium on Groundwater Flow and Transport Modelling. Proceedings of the Invited Lectures of Symposium on Groundwater Flow and Transport Modelling.

[31] Ministry of the Environment and Physical Planning (2000). National Environmental Action Programme.

[32] NLZOH, ZAG, NIJZ (2016). Priporočila za Ocenjevanje Primernosti Materialov in Proizvodov, ki Prihajajo v Stik s Pitno Vodo in so del Vodovodnega Omrežja in Interne Vodovodne Napeljave (P-MPPV).

[33] Novak Babič M., Gunde-Cimerman N. (2023). Culturable mycobiota of drinking water in Göteborg (Sweden) in comparison to Ljubljana (Slovenia) with implications on human health. J. Water Health.

[34] WHO (2010). Hardness in Drinking-Water Background Document for Development of WHO Guidelines for Drinking-Water Quality.

[35] Caubel-Forget V., Grimaldi C., Rouault F. (2001). Contrasted dynamics of nitrate and chloride in groundwater submitted to the influence of a hedge. Comptes Rendus L’Académie Sci. Ser. IIA Earth Planet. Sci..

[36] Ali E.A.M., Abdel-Rahman T.M.A., Sayed M.A.-E., Khale S.A.A.H. (2017). Occurrence of Fungi in Drinking Water Sources and Their Treatment by Chlorination and UV-Irradiation. Egypt. J. Bot..

[37] Ren W., Huang T., Wen G. (2023). Quantity, Species, and Origin of Fungi in a Groundwater-Derived Water Source. Water.

[38] Parveen S., Lanjewar S., Sharma K., Kutti U. (2011). Isolation of fungi from the surface water of river. J. Exp. Sci..

[39] Izah S.C., Richard G., Sawyer W.E. (2021). Distribution of Fungi density and diversity in a Surface water of Epie Creek in Yenagoa Metropolis, Nigeria. Arch. Epidemiol. Public Health.

[40] Heinrichs G., Hübner I., Schmidt K.C., de Hoog G.S., Haase G. (2013). Analysis of black fungal biofilms occurring at domestic water taps (I): Compositional analysis using Tag-encoded FLX amplicon pyrosequencing. Mycopathologia.

[41] Heinrichs G., Hübner I., Schmidt K.C., de Hoog G.S., Haase G. (2013). Analysis of black fungal biofilms occurring at domestic water taps (II): Potential routes of entry. Mycopathologia.

[42] Madrid H., Hernández-Restrepo M., Gené J., Cano J., Guarro J., Silva V. (2016). New and interesting chaetothyrialean fungi from Spain. Mycol. Progress..

[43] Göttlich E., van der Lubbe W., Lange B., Fiedler S., Melchert I., Reifenrath M., Flemming H.-C., de Hoog G.S. (2002). Fungal flora in groundwater-derived public drinking water. Int. J. Hyg. Environ. Health.

[44] Coleine C., Stajich J.E., de los Ríos A., Selbmann L. (2021). Beyond the extremes: Rocks as ultimate refuge for fungi in drylands. Mycologia.

[45] Pontara A.V., de Oliveira C.D., Barbosa A.H., Dos Santos R.A., Pires R.H., Martins C.H. (2011). Microbiological monitoring of mineral water commercialized in Brazil. Braz. J. Microbiol..

[46] WHO (2017). Guidelines for Drinking Water Quality.

[47] Kishanrao S. (2022). Beware! Fungus Growth in Reverse Osmosis Filters: On Exposure to Direct Sunlight. Acta Sci. Clin. Case Rep. ASCR.

[48] Pereira V.J., Marques R., Marques M., Benoliel M.J., Barreto Crespo M.T. (2013). Free chlorine inactivation of fungi in drinking water sources. Water Res..

[49] Pangloli P., Hung Y.-C. (2013). Effects of water hardness and pH on efficacy of chlorine-based sanitizers for inactivating Escherichia coli O157:H7 and Listeria monocytogenes. Food Control.

[50] Hurtado-McCormick S., Sánchez L., Martínez J., Calderón C., Calvo D., Narváez D., Lemus M., Groot H., Rodríguez Susa M. (2016). Fungi in biofilms of a drinking water network: Occurrence, diversity and mycotoxins approach. Water Supply.

[51] Zhu Y., Chen L., Xiao H., Shen F., Deng S., Zhang S., He J., Song C., Wang X., Zhang J. (2020). Effects of disinfection efficiency on microbial communities and corrosion processes in drinking water distribution systems simulated with actual running conditions. J. Environ. Sci. (China).

[52] Richardson M., Rautemaa-Richardson R. (2019). Exposure to Aspergillus in Home and Healthcare Facilities’ Water Environments: Focus on Biofilms. Microorganisms.

[53] DEFRA (Department for Environment, Food & Rural Affairs) (2011). A Review of Fungi in Drinking Water and the Implications for Human Health.

[54] Douterelo I., Dutilh B.E., Arkhipova K., Calero C., Husband S. (2020). Microbial diversity, ecological networks and functional traits associated to materials used in drinking water distribution systems. Water Res..

[55] Phatai P., Wittayakun J., Chen W.-H., Morales Futalan C., Grisdanurak N., Kan C.-C. (2014). Removal of manganese(II) and iron(II) from synthetic groundwater using potassium permanganate. Desalin. Water Treat..

[56] Gerrits R., Pokharel R., Breitenbach R., Radnik J., Feldmann I., Schuessler J.A., von Blanckenburg F., Gorbushina A.A., Schott J. (2020). How the rock-inhabiting fungus *K. petricola* A95 enhances olivine dissolution through attachment. Geochim. Cosmochim. Acta.

[57] Philippe A., Noël C., Eyheraguibel B., Briand J.-F., Paul-Pont I., Ghiglione J.-F., Coton E., Burgaud G. (2023). Fungal Diversity and Dynamics during Long-Term Immersion of Conventional and Biodegradable Plastics in the Marine Environment. Diversity.

[58] WHO (2022). Fungal Priority Pathogens List to Guide Research, Development and Public Health Action.

[59] de Hoog G.S., Guarro J., Gené J., Ahmed S., Al-Hatmi A.M.S., Figueras M.J., Vitale R.G. (2020). Atlas of Clinical Fungi.

[60] Mhlongo N.T., Tekere M., Sibanda T. (2019). Prevalence and public health implications of mycotoxigenic fungi in treated drinking water systems. J. Water Health.

